# Under the Arctic sun: assessment of skin cancer risk and sun-protection behaviours in Indigenous communities in Nunavik, an emerging at-risk population for skin cancers

**DOI:** 10.1093/bjd/ljaf290

**Published:** 2025-07-30

**Authors:** Alexandra S V Kelly, Amina Moustaqim-Barrette, Chenrui Xie, Arusa Shah, Angie Moshutz, Said Dababneh, Nadine Dababneh, Phedra Fadel, Agustina Hasbani, Sammy Pootoo, Putulik Ilisituk, Mary Sala, Parsa Kitishimik, Serena Weetaltuk, Annie Kumarluk, Richard Moorhouse, Martha Inukpuk, Sandra Peláez, François Lagacé, Ivan V Litvinov

**Affiliations:** St. Mary’s Research Centre, Montreal, QC, Canada; Faculty of Arts and Sciences, University of Ottawa, Ottawa, ON, Canada; St. Mary’s Research Centre, Montreal, QC, Canada; St. Mary’s Research Centre, Montreal, QC, Canada; St. Mary’s Research Centre, Montreal, QC, Canada; Research Centre of Sainte-Justine University Hospital, Montreal, QC, Canada; St. Mary’s Research Centre, Montreal, QC, Canada; St. Mary’s Research Centre, Montreal, QC, Canada; Ungava Tulattavik Health Centre, Kuujjuaq, QC, Canada; Inuulitsivik Health Centre, Puvirnituq, QC, Canada

## Abstract

Our work highlights important disparities in sun-protective behaviours and skin cancer awareness/knowledge gaps between Inuit Indigenous vs. general Canadian populations. Our work identifies multiple concerns and lack of sun protection that could lead to preventable death and suffering from skin cancers in this emerging at-risk population.

Dear Editor, The Inuit Nunangat region is home to > 49 000 Inuit and encompasses four territories in Canada’s circumpolar region: the Inuvialuit Settlement Region, Nunavut, Nunavik and Nunatsiavut.^1^ Despite traditional perceptions of lower risk attributed to the darker skin tone of the Inuit,^2^ residents experience prolonged summer daylight, high levels of albedo – the fraction of light/ultraviolet radiation (UVR) reflected off surfaces – from snow and ice, and minimal natural shade. In addition, the region relies on nurse and allied health workers as primary healthcare providers, with only sporadic specialist access available to these remote and isolated Northern communities. Our group has previously evaluated sun-protection behaviours and skin cancer knowledge in several Canadian regions.^3,4^ Given the unique environment in Nunavik, varying perceptions on UVR exposure, and changing climate that is accelerated in the Arctic, we sought to understand sun-protection practices and identify gaps in skin cancer knowledge among Inuit living in Nunavik. While darker skin pigmentation offers partial protection from UVR-induced damage, Indigenous groups, including those in high-risk environments, can develop melanoma and other skin cancers at significant rates. For example, New Zealand’s Māori population had an age-standardized incidence rate (ASIR) of 34.6 cases per 100 000 in 2006,^5^ substantially higher than Canada’s 2011–2017 melanoma ASIR of 14.12 per 100 000.^3,4^ Consistent with this data, recent reports highlight that skin cancers have significant impact on American Indian/Alaska Native Communities, where it was documented that these populations endure the second highest rate of melanoma in the USA.^6^

To collect data on UVR exposure, sun-protective habits and skin cancer knowledge, we administered the ­cross-sectional Sun Exposure and Behaviour Inventory (SEBI) across four Nunavik communities between May and August 2024. A total of 541 Inuit participants aged ≥ 16 years completed the electronic survey (in-person with support from our research staff working in Nunavik communities). Results were compared with data from two prior SEBI surveys of general populations in Manitoba (*n* = 3347) and Atlantic Canada (*n* = 7861) collected by our team.

Total lifetime sun exposure was lower in Nunavik (56.3%) vs. 85.2% in Atlantic Canada and 87.8% in Manitoba (Figure 1). While occupational sun exposure was more common in Nunavik (Nunavik, 27.4%; Atlantic, 10.9%; Manitoba, 12.0%), recreational sun exposure was less frequent (Nunavik, 59.4%; Atlantic, 72.8%; Manitoba, 77.5%). The proportion of participants reporting more than 10 lifetime sunburns was lower in Nunavik (18.5%), compared with 69.7% in Atlantic Canada and 71.0% in Manitoba.

**Figure 1:**
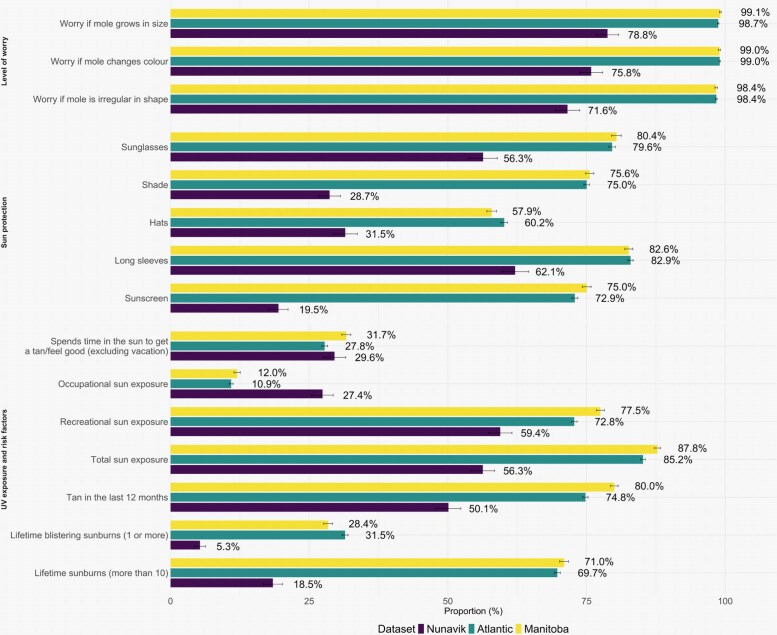
Comparison of sun exposure, melanoma risk factors, sun-protection habits and level of worry for skin changes across Nunavik, Atlantic Canada and Manitoba datasets.

Use of common sun-protection measures was significantly reduced in Nunavik. Only 28.7% of respondents in Nunavik reported always or almost always seeking shade, vs. ∼75% in both comparison groups. Similarly, hat use was reported by 31.5% in Nunavik, vs. ∼60% in both comparator groups. Sunscreen use was notably low in Nunavik (19.5%) compared with over 72% in Atlantic and Manitoba populations. Wearing long-sleeved clothes was the most comparable measure (Nunavik, 62.1%; Atlantic, 82.9%; Manitoba, 82.6%).

Awareness concerning mole changes was also markedly lower. While over 98% of Manitoba and Atlantic respondents were concerned about changes in mole size, colour or shape, only 78.8%, 75.8% and 71.6% of Nunavik participants, respectively, shared these concerns.

Significant health inequities persist between Canada’s Indigenous and non-Indigenous communities; on average, Indigenous Peoples in Canada live 8.9–9.6 years fewer than non-Indigenous Canadians.^7^ While this study generally corroborates our group’s data evaluating UVR exposure and sun-protection practices from other Canadian cohorts,^4,5,8^ it highlights important disparities between Inuit and general Canadian populations. The identified gap is most likely multifactorial, and could be related to environmental factors, varying perception of risk related to sun protection/skin cancer, and socioeconomic, cultural and geographical barriers. A lack of resources and support for sun-protection products may also add to the lack of sun-protective practice in northern communities. Indeed, as there is no subsidy on ­sun-protection products, a potential update to the Nutrition North Canada programme could consider including subsidies for sunscreen alongside diapers and menstrual products as part of the nonfood items. Future research should identify actionable culturally sensitive interventions from the perspective of Inuit individuals.

Our use of a validated survey tool allowed for robust data collection, as well as comparison with previous cohorts for contextualization of our findings. Limitations include possible selection and recall bias. Differences in educational access and historical context across regions may have influenced participants’ interpretations of survey questions, and thus comparisons between cohorts (e.g. Nunavik, Atlantic Canada and Manitoba) should be made with caution. In addition, some bias may have been introduced due to translation errors or misunderstanding of terms more commonplace in Canadian urban communities. Finally, few data exist on the predominant types of melanoma affecting Northern Indigenous Canadian populations, and the possibility exists that non-UVR-dependent melanoma (including acral lentiginous melanoma) is of greater concern in these populations. Nevertheless, as highlighted by the US study,^6^ rates of melanoma/skin cancers in Alaska Native and Canadian native communities deserve more consideration. Furthermore, data from New Zealand have demonstrated a much greater proportion of thick melanomas (>  4-mm thick) in dark-skinned Māori, Pacific and Asian New Zealanders compared with white-skinned New Zealanders,^5^ which highlights the need for further research on melanoma in Indigenous communities.

This study provides valuable insights into the ­sun-protection behaviours, UVR exposure patterns, and skin cancer risk perceptions of Inuit communities in Nunavik, while highlighting disparities when compared with general populations in Canada. Our findings suggest opportunities to develop culturally sensitive and relevant, community-driven strategies to address persistent health gaps in Canada and may inform future research directions in Canada and beyond.

## Supplementary Material

ljaf290_Supplementary_Data

## Data Availability

The data underlying this article will be shared on reasonable request to the corresponding author.
