# Supplementary material for: Under the Arctic sun: assessment of skin cancer risk and sun-protection behaviours in Indigenous communities in Nunavik, an emerging at-risk population for skin cancers
Source: Br J Dermatol. 2025 Jul 30;193(5):1021–4. doi: 10.1093/bjd/ljaf290 (PMC12532138; doi:10.1093/bjd/ljaf290)
Supplement: ljaf290_Supplementary_Data [file ljaf290_supplementary_data.docx]

**Appendix S1**  Complete list of author affiliations.

Alexandra S V Kelly,^1,2^ Amina Moustaqim-Barrette,^1,3^ Chenrui Xie,^1,3^ Arusa Shah,^4^ Angie Moshutz,^3^ Said Dababneh,^4^ Nadine Dababneh,^4^ Phedra Fadel,^3^ Augustina Hasbani,^1^ Sammy Pootoo,^5^ Putulik Ilisituk,^5^ Mary Sala,^6^ Parsa Kitishimik,^6^ Serena Weetaltuk,^6^ Annie Kumarluk,^7^ Richard Moorhouse,^8^ Martha Inukpuk,^8^ Sandra Peláez,^9,10^ François Lagacé^1^ and Ivan V Litvinov^1,10,11,12^

^1^St. Mary’s Research Centre, Montreal, QC, Canada

^2^Faculty of Arts and Sciences, University of Ottawa, Ottawa, ON, Canada

^3^Faculty of Medicine and Health Sciences, McGill University, Montreal, QC, Canada

^4^Faculty of Medicine, University of Montreal, Montreal, QC, Canada

^5^Municipality of Salluit, Salluit, QC, Canada

^6^Municipality of Kuujjuaraapik, Kuujjuaraapik, QC, Canada

^7^Municipality of Kuujjuaq, Kuujjuaq, QC, Canada

^8^Municipality of Inukjuak, Inukjuak, QC, Canada

^9^School of Kinesiology and Physical Activity Sciences, Faculty of Medicine, University of Montreal, Montreal, QC, Canada

^10^Research Centre of Sainte-Justine University Hospital, Montreal, QC, Canada

^11^Ungava Tulattavik Health Centre, Kuujjuaq, QC, Canada

^12^Inuulitsivik Health Centre, Puvirnituq, QC, Canada
